# ﻿*Didymodonmanhanensis* (Pottiaceae, Bryophyta), a new species from Inner Mongolia steppe, China and its phylogenetic position, based on molecular data

**DOI:** 10.3897/phytokeys.197.80531

**Published:** 2022-05-23

**Authors:** Chao Feng, Guo-Li Zhang, Ting-Ting Wu, Jin Kou

**Affiliations:** 1 College of Grassland, Resources and Environment, Key Laboratory of Grassland Resources, Ministry of Education China, Key Laboratory of Forage Cultivation, Processing and High Efficient Utilization of Ministry of Agriculture, Inner Mongolia Agricultural University, Hohhot 010011, China. Inner Mongolia Agricultural University Hohhot China; 2 School of Life Sciences, Key Laboratory of Molecular Epigenetics of Ministry of Education, Key Laboratory of Vegetation Ecology, Ministry of Education, Northeast Normal University, Changchun 130024, China Northeast Normal University Changchun China

**Keywords:** Asia, Manhan Mountain, phylogenetic analysis, taxonomy

## Abstract

Inner Mongolia steppe is one of the suitable habitats for *Didymodon* species and a new species, *Didymodonmanhanensis* C. Feng & J. Kou from Manhan Mountain in semi-arid region in Inner Mongolia, China is described and illustrated. It is characterised by leaves incurved and slightly twisted when dry, spreading when moist, narrowly lanceolate from an ovate base; subulate and fragile leaf apices; distally bistratose leaf margins that are recurved in proximal 2/3–3/4; excurrent costa with guide cells in 2–3 layers and without ventral stereids; smooth laminal cells and red KOH laminal colour reaction. Our morphological analyses and molecular results, based on DNA sequences of ITS, *rps*4 and *trn*M-*trn*V, conﬁrm that *D.manhanensis* belongs to a group that includes *D.obtusus* J. Kou, X.-M. Shao & C. Feng and *D.daqingii* J. Kou, R.H. Zander & C. Feng. This new species is compared with similar species and its phylogenetic position and ecology are discussed.

## ﻿Introduction

Inner Mongolia, situated in Inner Eurasia, is located in the northern part of China and presents a strip distribution from the northeast to the west. The district habitats are temperate continental monsoon climate. The annual mean temperature is 8 °C, which increases from east to west and the annual precipitation is 35–530 mm, which decreases from southeast to northwest (Miao 2017). The area of grassland accounts for 60% of the whole Inner Mongolia and more than one-quarter of the total area of grassland in China. The grassland in Inner Mongolia is divided into three types: meadow steppe formed by, for example, *Stipabaicalensis* Roshev, *Leymuschinensis* (Trin.) Tzvelev; typical steppe formed by, for example, *Stipagrandis* P. Smirn., *Stipakrylovii* Roshev, *Leymuschinensis* (Trin.) Tzvelev and desert steppe formed by, for example, *Stipaklemenzii* Roshev, *Stipaglareosa* P.A. Smirn., *Stipabreviflora* Griseb (Hua et al. 2021). The main vegetation types of the steppes present distinct zonal features (Wu et al. 2005). The Inner Mongolia steppe is a suitable habitat for the *Didymoodn* Hedw. species and several new species were recently discovered (e.g. Kou et al. 2016a; Kou et al. 2019; Feng et al. 2022).

The taxonomy of genus *Didymodon* is complicated, involving the differentiation from related genera, such as *Barbula* Hedw. and the circumscriptions of its infrageneric sections (Zander 1993; Zander 2007; Zhang et al. in press). A recent important event associated with *Didymodons. lat.* was the split of the genus into seven smaller genera: *Aithobryum* R.H. Zander, *Didymodons. str.*, *Exobryum* R.H. Zander, *Fuscobryum* R.H. Zander, *Geheebia* Schimp., *Trichostomopsis* Card. and *Vinealobryum* R.H. Zander, based on macro-evolutionary analysis and the dissilient genus concept applied (Zander 2013; Zander 2019). Although initially this revolutionary concept was considered unnecessary or unsupported (Blockeel and Kučera 2019), it has later been supported by molecular phylogenetic data, but with some alterations (Jiménez et al. 2022; Zhang et al. in press) and has gained acceptance by some other authors (e.g. Kou and Feng 2018; Osman et al. 2021; Feng et al. 2022). During our continuous investigations of xerophilic mosses, especially Pottiaceae Hampe, in China (e.g. Feng et al. 2016a, b; Kou et al. 2016b, 2018, 2019; Kou and Feng 2018), many specimens have been collected from different provinces. Amongst them, two samples collected from Manhan Mountain in Inner Mongolia of *Didymodon* s. lat. from stony habitats are different from species previously reported in the area (Li et al. 2001). They mostly resemble *Didymodonobtusus* J. Kou, X.-M. Shao & C. Feng. To clarify their taxonomic identity, we conducted phylogenetic analysis and confirm that these samples belong to the genus *Didymodons. str.* (Zander 2013), but do not match with any species known in the genus. Here, we describe this unknown moss as a new species.

## ﻿Materials and methods

### ﻿Morphological observations

Over 3000 specimens of the genus *Didymodons. lat.* were examined during our revision of Pottiaceae in China. More than 50 field investigations were conducted in past years and the specimens included in this study were housed in the Herbaria at IFP, KUN and NMAC. Microscopic examinations and measurements were taken with a ZEISS Primo Star light microscope and photomicrographs were obtained with a Canon EOS 70D camera, mounted on this microscope. Specimens were examined in 2% potassium hydroxide (KOH). Three plants were dissected from each collection and, for each shoot, every possible structure from the gametophyte had to be examined and a record kept of what was found for each individual species. Specific morphological and anatomical features of taxonomic importance were assessed mainly following Zander (1993). Leaves were always taken from the upper and middle parts of the stem and cross-sections were made in the middle part of the stem. Measurements of leaf width were taken at the base, mid-leaf and upper part. Cross-sections were made at mid-leaf.

### ﻿Phylogenetic analyses

To test the phylogenetic position of the new species, two specimens collected from Manhan Mountain were sampled. Due to its great similarity with *D.obtusus* and *Didymodondaqingii* J. Kou, R.H. Zander & C. Feng, the isotypes of the two species were added to the dataset. We employed one nuclear (ITS) and two chloroplast markers (*rps*4 and *trn*M-*trn*V), which had been used successfully in previous phylogenetic studies in *Didymodon* s. lat. and enabled the re-use of earlier results and easier interpretation of new data (Werner et al. 2004, 2005, 2009; Kučera and Ignatov 2015; Kučera et al. 2018; Ronikier et al. 2018; Jiménez et al. 2022; Zhang et al. in press). Phylogenetic trees are created and shown separately. The complete list with sample names and GenBank accession numbers is presented in Tables 1 and 2. DNA extraction, PCR ampliﬁcation and sequencing procedure followed the protocols described by Wang et al. (2010).

**Table 1.** New sequences used in this study, including taxa vouchers information and GenBank accession numbers.

Species	Voucher information	ITS	*rps*4	*trn*M-*trn*V
*Didymodonmanhanensis 4*	China, Inner Mongolia, Chao Feng 2016060162	OL514237	OL450506	OL450515
*Didymodonmanhanensis a3*	China, Inner Mongolia, Chao Feng 2016060176	OL514238	OL450507	OL450516
*Didymodonobtusus*	China, Tibet, Xiao-Ming Shao & Jin Kou 20140815037	OL514239	OL450508	OL450517
*Didymodondaqingii*	China, Inner Mongolia, Chao Feng 20170605032	OL514240	OL450509	OL450518

**Table 2:** . Sequences from GenBank used in this study, including taxa and GenBank accession numbers.

Species	ITS	*rps4*	*trnM-trnV*
*Acaulontriquetrum*	MW398556		
*Aloinarigida*	MW398549		
*Aloinellaandina*	MW398550		
*Andinellachurchilliana*	MW398720		
*Andinellacoquimbensis*	MW398711		
*Andinellaelata*	MW398708		
*Andinellagranulosa*	MW398714		
*Andinellalimensis*	MW398710		
*Andinellaoedocostata*	MW398733		
*Andinellapruinosa*	MW398726		
*Barbulaunguiculata*	MW398553	HM147777	JQ890366
*Bryoerythrophyllumrecurvirostrum*	MW398547	JQ890468	JQ890407
*Bryoerythrophyllumrubrum*	MW398548		
*Chenialeptophylla*	MW398561		
*Cinclidotusriparius*	MW398554		
*Crossidiumsquamiferum*	MW398558		
*Didymodonacutus*	AY437111	KP307551	KP307667
*Didymodonalpinus*	MW398606		
*Didymodonandreaeoides*	MW398768		
*Didymodonanserinocapitatus*	MW398649	KP307545	KP307640
*Didymodonasperifolius*	MW398594	JQ890472	KP307600
*Didymodonaustralasiae* (*Trichostomumaustralasiae*)	MW398737	KP307571	KP307651
*Didymodonbrachyphyllus* (*Vinealobryumbrachyphyllum*)	MW398817		
*Didymodonbuckii*	MW398578		
*Didymodoncaboverdeanus*	MW398607		
*Didymodoncalifornicus* (*Vinealobryumcalifornicum*)	MW398819		
*Didymodoncanoae*	MW398584		
*Didymodoncardotii*	MW398729		
*Didymodonchallaensis* (*Trichostomopsischallaensis*)	MW398748		
*Didymodonconstrictus*	MW398613		
*Didymodoncordatus*	MW398664	KP307564	KP307668
*Didymodonditrichoides*	MW398642		
*Didymodoneckeliae* (*Vinealobryumeckeliae*)	MW398826		
*Didymodonedentulus*	MW398685		
*Didymodonepapillatus*	MW398665		
*Didymodonerosodenticulatus*	MW398792	MF536597	MF536635
*Didymodonerosus*	EU835148	MF536609	MF536646
*Didymodonfallax* (*Geheebiafallax*)	MW398779	KP307552	KP307663
*Didymodonferrugineus* (*Geheebiaferruginea*)	MW398796	MF536588	MF536625
*Didymodonfragilicuspis*	KP307482		
*Didymodonfuscus*	MW398689	KP307537	KP307601
Didymodonaff.fuscus		KP307546	KP307615
*Didymodongaochienii*		KP307538	KP307658
*Didymodongelidus*	MW398693		
*Didymodongiganteus*	MW398786	KP307548	KP307669
*Didymodonglaucus*	MW398612		
*Didymodonguangdongensis* (*Vinealobryumguangdongense*)	MW398657		
*Didymodonhedysariformis*	MW398582	KP307569	KP307629
*Didymodonhengduanensis*	MW398629		
*Didymodonhegewaldiorum*	MW398739		
*Didymodonherzogii*	MW398746		
*Didymodonhumboldtii*	MW398667		
*Didymodonicmadophilus*	MW398632	KP307598	KP307604
*Didymodonimbricatus*	MW398646		
*Didymodonincrassatolimbatus*	MW398572		
*Didymodonincurvus*	MW398680		
*Didymodoninsulanus* (*Vinealobryuminsulanum*)	MW398811		
*Didymodonjaponicus*	MW398757		
*Didymodonjimenezii*	MW398622		
*Didymodonjohansenii*	MW398589	KP307542	KP307662
*Didymodonkunlunensis*	MW398610		
*Didymodonlaevigatus*	MW398618		
*Didymodonlainzii*	MW398575		
*Didymodonleskeoides* (*Geheebialeskeoides*)	MW398777	MF536604	MF536642
*Didymodonluehmannii*	MW398718		
*Didymodonluridus*	AY437098	MF536587	MF536624
*Didymodonmaschalogena*	MW398615		
*Didymodonmaximus* (*Geheebiamaxima*)	MW398784	MF536591	MF536628
*Didymodonmesopapillosus*	MW398758		
*Didymodonmolendoides*	MW398687		
*Didymodonmongolicus*	KU058175		
*Didymodonmurrayae*	KP307513	KP307563	KP307650
*Didymodonnevadensis*	MW398730		
*Didymodonnicholsonii* (*Vinealobryumnicholsonii*)	MW398808		
*Didymodonnigrescens*	LC545516	KP307543	KP307611
*Didymodonnorrisii*	MW398830	KP307585	KP307617
*Didymodonnovae-zelandiae*	MW398769		
*Didymodonobtusus*	MW398666		
*Didymodonoccidentalis*		KP307533	KP307599
*Didymodonochyrarum*	MW398763		
*Didymodonparamicola* (*Trichostomopsisparamicola*)	MW398740		
*Didymodonpatagonicus*	MW398675		
*Didymodonperobtusus*	KP307523	KP307539	KP307609
*Didymodonrevolutus* (*Husnotiellarevoluta*)	MW398569	JQ890471	KP307646
Didymodonrevolutusvar.africanus	MW398568		
*Didymodonrigidulus*	MW398602	KP307589	KP307647
Didymodonrigidulusvar.subulatus	MW398672		
*Didymodonrivicola*	MW398599	KP30756	KP307607
*Didymodonsantessoni*	MW398705		
*Didymodonsicculus*	MW398801	MF536606	MF536643
*Didymodonsinuosus*	MW398567	JQ890476	JQ890410
*Didymodonspadiceus* (*Geheebiaspadicea*)	MW398795	MF536593	MF536631
*Didymodonsubandreaeoides*	AY437108	KP307570	KP307630
*Didymodontectorum*	MW398659		
*Didymodontibeticus*	MW398638		
*Didymodontomaculosus*	AY437114		
*Didymodontophaceus*	MW398807	MF536607	MF536644
Didymodontophaceusvar.anatinus		MF536589	MF536626
*Didymodontorquatus*	MW398719		
*Didymodonumbrosus* (*Trichostomopsisumbrosa*)	MW398742		
*Didymodonvalidus*	MW398650		
*Didymodonvinealis* (*Vinealobryumvineale*)	MW398815	JQ890475	KP307606
Didymodonvinealisvar.rubiginosus	MW398822		
*Didymodonvulcanicus*	MW398636		
*Didymodonwaymouthii*	MW398770		
*Didymodonwisselii*	MW398655		
*Didymodonxanthocarpus*	MW398696	KP307534	KP307638
*Didymodonzanderi*	MW398585	KP307535	KP307621
*Dolotortulamniifolia*	MW398555		
*Erythrophyllopsisandina*	MW398546		
*Gertrudiellauncinicoma*	MW398698		
Gertrudiellauncinicomavar.serratopungens	MW398701		
*Guerramontesiamicrodonta*	MW398543		
*Hennediellaheimii*	GQ339750		
*Hennediellapolyseta*	GQ339759		
*Leptodontiumexcelsum*	MW398545		
*Microbryumcurvicolle*		JX679986	JX679936
*Microbryumdavallianum*	MW398557		
*Pseudocrossidiumhornschuchianum*	MW398551	JQ890481	JQ890420
*Pseudocrossidiumrevolutum*	MW398552		
*Pterygoneurumovatum*	MW398560		
*Sagenotortulaquitoensis*	GQ339761		
*Stegonialatifolia*	MW398559		
*Syntrichiaruralis*	MW398564	FJ546412	FJ546412
*Tortulamuralis*	MW398562	JN581679	JQ890421
*Tortulasubulata*	MW398563		
*Triquetrellaarapilensis*	MW398544		
*Tridontiumtasmanicum*	MW398750		

The sequences were aligned by using MAFFT 7.222 (Kazutaka and Daron 2013) and then edited in BioEdit 7.0.1 (Hall 1999). The concatenation of individual *rps*4 and *trn*M-*trn*V fragments was performed by our custom Perl script. Phylogenetic analyses were performed by using the Bayesian Inference (BI) and Maximum Likelihood (ML) methods. MrBayes 3.2.6 (Ronquist et al. 2012) was used for BI analyses under the GTR substitute model. The following was used: two Markov Chain Monte Carlo (MCMC) searches were run for 10 million generations each, with a sampling frequency of 1000. The first 25% of the trees were discarded as burn-in. The convergence between runs in all cases dropped below 0.01. ML analyses were executed in IQ-TREE 1.6.3 (Nguyen et al. 2014) under the TPM3u+F+R3 (for cpDNA) and TIM3e+I+G4 (for ITS) substitute models, respectively, selected by the ModelFinder programme (Kalyaanamoorthy et al. 2017), based on the Bayesian Information Criterion (BIC) and 1000 fast bootstrapping replicates were used. The final obtained trees were visualised and edited in FigTree v.1.4.0 (Rambaut 2014).

## ﻿Results

The chloroplast (cp) and ITS alignments comprised 1313 and 1364 nucleotide sites, respectively. The BI and ML phylogenetic trees have a consistent topology, although there are different levels of support depending on the method. Hence, only the topologies with branch lengths from the BI trees are presented, with added support from the ML method on the respective trees (Figs 3–4). Although the inference from analysed chloroplast regions (Fig. 3) and the ITS (Fig. 4) agrees in most aspects, the position of the new species is different between the two above phylogenetic trees and, thus, both of them are reserved. The topology of the ITS dataset shows that *D.manhanensis* is nested within the monophyletic group comprising *Didymodonepapillatus* J. Kou, X.-M. Shao & C. Feng, *Didymodonmongolicus* D.-P. Zhao & T.-R. Zhang, *Didymodonvalidus* Limpr., *Didymodonwisselii* (Dixon) D.H. Norris & T.J. Kop. and *Vinealobryumguangdongensis* C. Feng & J. Kou and is sister to *D.obtusus*, but with weakly-supported values. In the combined plastid dataset, *D.manhanensis* is nested within the group including *Didymodoncordatus* Jur. and is sister to *D.daqingii* and *Didymodonanserinocapitatus* (X.J. Li) R.H. Zander, with well-supported values.

## ﻿Discussion

As indicated by Zander (1993), *Didymodons. lat.* is heterogeneous and could be profitably split. In our phylogenetic analyses, this genus is polyphyletic and its species can be classified within several well-supported monophyletic clades, which correspond to other phylogenetic studies of the genus (Feng et al. 2022; Jiménez et al. 2022; Zhang et al. in press). Our results reveal a close relationship between *D.manhanensis* and two recently-described species in China: *D.daqingii* and *D.obtusus*. Although the latter two species were considered identical by Sollman et al. (2020), they are not closely related in our phylogenetic analyses, based on both ITS and chloroplast data.

*Didymodonmanhanensis* is distinguished from all congeners by the following combination of diagnostic features: leaves incurved and slightly twisted when dry, spreading when moist, narrowly lanceolate from an ovate base; subulate and fragile leaf apices; distally bistratose leaf margins that are recurved in proximal 2/3–3/4; costal guide cells in 2–3 layers and without ventral stereids, smooth laminal cells and red KOH laminal colour reaction. This combination of characters suggests the placement of *D.manhanensis* in the sect.Didymodon (Zander 1978, 1993, 1998). Following the recent revolutionary work on the genus *Didymodons. lat.* by Zander (2013, 2019), morphologically, it belongs in the amended genus *Didymodons. str.* Its systematic position in *Didymodons. str.* was also confirmed by our phylogenetic analyses, based on both ITS and chloroplast data.

Chloroplast data support that *D.manhanensis* is closely related to *D.cordatus* and sister to both *D.daqingii* and *D.anserinocapitatus*. However, *D.manhanensis* differs morphologically from *D.cordatus* by the costa with guide cells in 2–3 layers and without ventral stereids and smooth laminal cells. It differs from *D.daqingii* by the leaves that are narrowly lanceolate from an ovate base, smooth laminal cells and red KOH laminal colour reaction; it differs from *D.anserinocapitatus* by the distally bistratose leaf margins and lack of swollen and deciduous leaf apex (Zander 2007). In the ITS analyses, there is successive branching of clades, including *D.obtusus* J. Kou, X.-M. Shao & C. Feng, *D.manhanensis*, *D.epapillatus* J. Kou, X.-M. Shao & C. Feng, *D.mongolicus* D.-P. Zhao & T.-R. Zhang, *D.validus* Limpr., *Vinealobryumguangdongensis* C. Feng & J. Kou and *D.wisselii* (Dixon) D.H. Norris & T.J. Kop. Amongst these species, *D.manhanensis* is most similar to *D.obtusus*, a species that was recently described from Tibet in China (Kou et al. 2018), but the former can be distinguished from the latter by its narrowly lanceolate leaves from an ovate base and spreading when moist, subulate and somewhat fragile leaf apex and unistratose distal lamina.

There are three species distributed in China that have excurrent costa and smooth laminal cells may be confused with the new species. *Didymodonditrichoides* (Broth.) X.-J. Li & S. He, a species known from North American, Asia (China) and the Atlantic Islands (Iceland) (Li et al. 2001; Zander 2007), differs from the new species by the unistratose leaf margins, costa with 1–2 layers of guide cells and with 0–1 layer of ventral stereids and yellowish KOH laminal colour reaction (Zander 2007). *Didymodonvalidus* Limpr. can be separated from *D.manhanensis* by the twisted and incurved leaves when dry, unistratose leaf margins, costa with 1 layer of guide cells and with 1–3 layers of ventral stereids and yellowish-green KOH laminal colour reaction (Shuayib et al. 2017).

The lanceolate to long-lanceolate leaves with a widely ovate base, distally bistratose leaf margins, excurrent costa and epapillose laminal cells are likewise found in *Didymodonochyrarum* J.A.Jiménez & M.J.Cano, a species described from tropical South America (Jiménez and Cano 2019), which may be confused with the new species. However, *D.ochyrarum* can be separated from *D.manhanensis* by its plane leaf margins, marginal basal cells running up the margin forming a distinctly differentiated area of transversely thick-walled cells and yellowish KOH laminal colour reaction.

### ﻿Taxonomic treatment

#### ﻿ Didymodon manhanensis

Taxon classificationPlantaePottialesPottiaceae

C. Feng & J. Kou sp. nov.

70A98184-1F76-5333-8D1C-D128E33BD3A0

**Figure 1.**
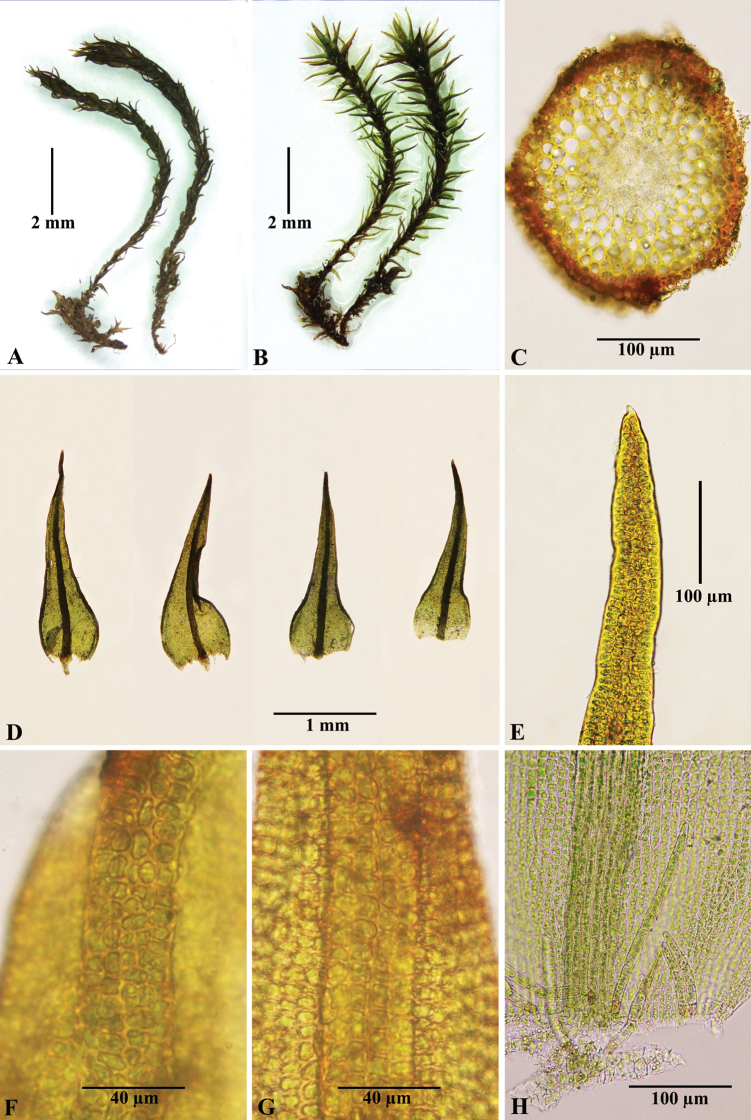
*Didymodonmanhanensis***A** dry plants **B** moist plants **C** cross-section of stem **D** leaves **E** leaf apex **F** upper part of costa (dorsal) **G** upper part of costa (ventral) **H** axillary hairs. Photographed on 21 November 2021 by Chao Feng from the holotype (NMAC!).

**Figure 2.**
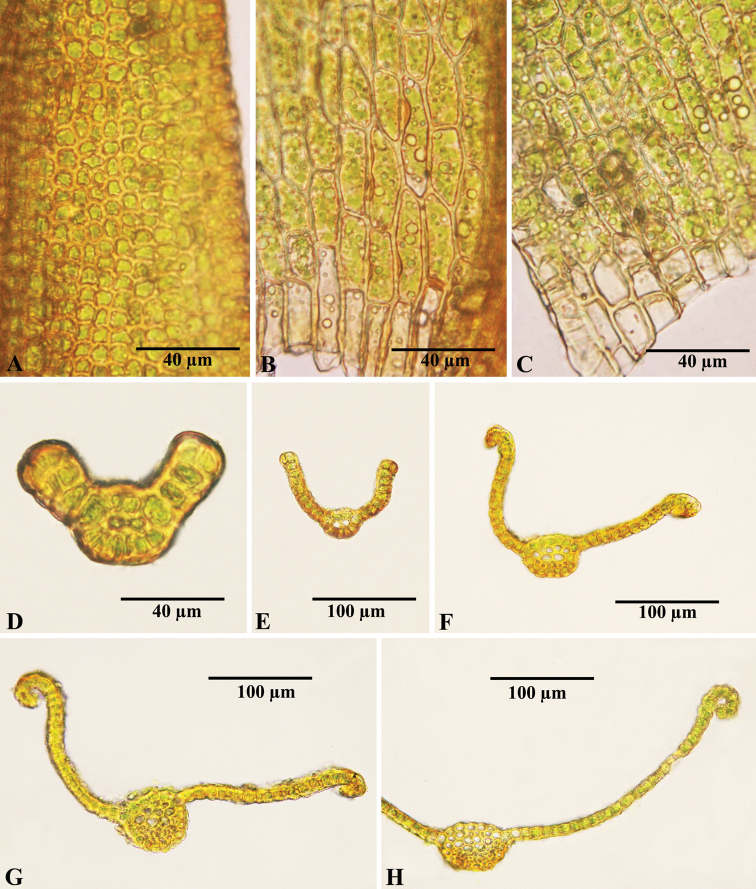
*Didymodonmanhanensis***A** median leaf cells **B** basal juxtacostal cells **C** basal marginal cells; **D–H** cross-sections of leaves, sequentially from apex to base. Photographed on 21 November 2021 by Chao Feng from the holotype (NMAC!).

Figs 1 , 2 Chinese name: 蛮汉山对齿藓

##### Type.

China. Inner Mongolia: Ulanqab City, Manhan Mountain, 40°39'19.2931"N, 112°19'36.3792"E, on soil under the grass, elevation 1417 m, 20 June 2016, *Chao Feng 2016060162* (holotype: NMAC!; isotype: MO!).

##### Diagnosis.

It is distinguished from all congeners by the following combination of diagnostic features: leaves incurved and slightly twisted when dry, spreading when moist, narrowly lanceolate from an ovate base; subulate and fragile leaf apices; distally bistratose leaf margins that are recurved in proximal 2/3–3/4; costal guide cells in 2–3 layers and without ventral stereids, smooth laminal cells and red KOH laminal colour reaction.

##### Description.

Plants medium, growing in turfs, green-blackish distally, brown-blackish proximally. Stems very seldom branched, 0.8–1.6 cm in length, not papillose, transverse section rounded to rounded-pentagonal, central strand developed, sclerodermis present, hyalodermis absent; axillary hairs filiform, of 4–8 hyaline cells, the basal cell brown. Leaves crowded on stem, incurved and slightly twisted when dry, spreading when moist, narrowly lanceolate from an ovate base, constricted just above the base, 1.3–2.3 × 0.43–0.55 mm, distal lamina narrowly channelled ventrally; margins plane distally, recurved in proximal 2/3–3/4 of leaf, entire, distal margins bistratose; apex subulate, somewhat fragile; leaf base ovate, not sheathing, not decurrent; costa stout, tapering distally, 57.5–75 µm wide at base, excurrent as a long, thick subula, not spurred, ventral cells of costa in upper middle part of leaf quadrate or subquadrate, smooth, 4 rows of cells across costa ventrally at mid-leaf, dorsal cells of costa in upper middle part of leaf quadrate or subquadrate, smooth, transverse section semicircular to nearly rounded, epidermis present adaxially and abaxially, not or weakly bulging, ventral stereids absent, guide cells 10–16 in 2–3 layers, 2–4 layers of dorsal stereids, reniform or crescent-shaped, without hydroids; upper laminal cells quadrate to rhombic, usually with angular lumens, 7.5–10 × 5–10 µm, smooth, slightly thick-walled, weakly convex on both surfaces, distal lamina unistratose, basal cells weakly differentiated juxtacostally, rectangular, 12.5–37.5 × 5–7.5 µm, thin-walled, smooth; basal marginal cells subquadrate or quadrate, 5–8.75 × 6.25–7.5 µm, with weakly-thickened walls, smooth. Gemmae absent. Dioicous. Sporophytes unknown. KOH laminal colour reaction red.

##### Additional specimens examined.

**China Inner Mongolia**: Ulanqab City, Manhan Mountain, on soil under the grass, 20 June 2016, Chao Feng 2016060176 (NMAC).

##### Etymology.

The specific epithet refers to Manhan Mountain, the type locality.

##### Habitat and distribution.

Manhan Mountain is situated in Liangcheng County in the southern section of the Yinshan Mountains in the middle of Inner Mongolia, with an average altitude of approximately 1500 m (Huang et al. 2014). Its soil types are mainly leaching grey, cinnamonic soil (Lyu et al. 2012). The vegetation on Manhan Mountain is typical forest shrub vegetation, including natural forest that consists of *Betulaplatyphylla* Sukaczev and *Populusdavidiana* Dode, plantation that consists of *Larixprincipis-rupprechtii* Mayr and Pinussylvestrisvar.mongolica Litv., natural shrubs that consists of *Ostryopsisdavidiana* Decne., *Spiraeasalicifolia* L. and *Rosadavurica* Pall. and the herbaceous plants including *Stipabungeana* Trin., *Cleistogenessquarrosa* (Trin.) Keng, *Lespedezabicolor* Turcz., *Carex* spp. and *Leymuschinensis* (Trin.) Tzvelev (Zhang et al. 2017; Li et al. 2021). *Didymodonmanhanensis* is currently known only from the type locality at the foot of the Manhan Mountain, north-western Liangcheng County, Inner Mongolia, China, growing on soil under the grass.

**Figure 3.**
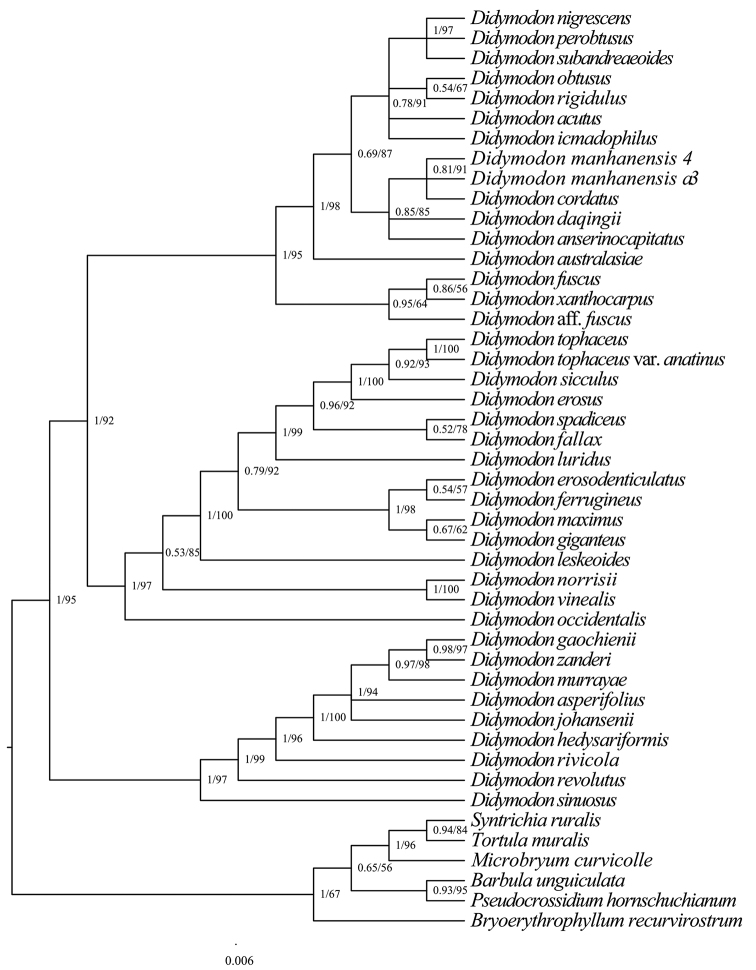
Phylogenetic relationships (50% majority consensus tree) from the Bayesian Inference of the concatenated *rps*4 and *trn*M-*trn*V datasets. Numbers above branches indicate posterior probability from the BI analysis, followed by bootstrap values for the ML analysis.

**Figure 4.**
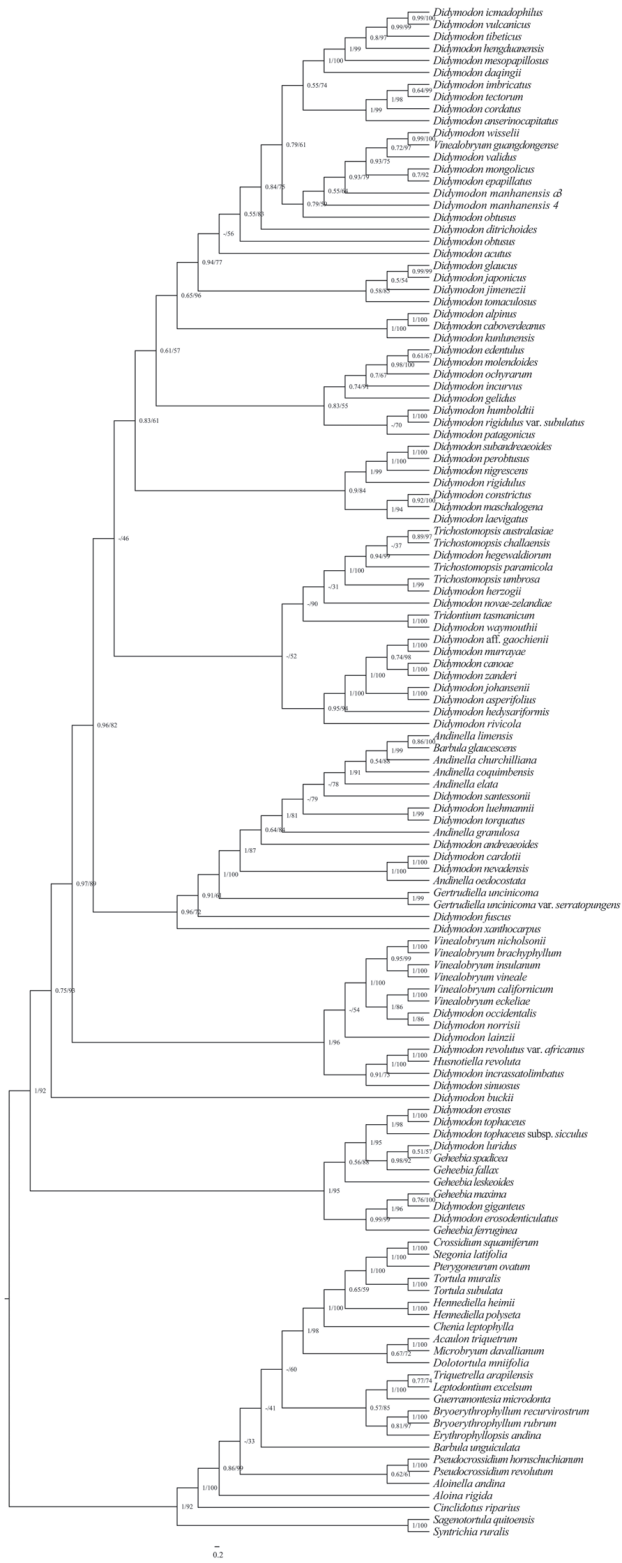
Phylogenetic relationships (50% majority consensus tree) from the Bayesian Inference on the ITS dataset. Numbers above branches indicate posterior probability from the BI analysis, followed by bootstrap values for the ML analysis.

### ﻿Key to species morphologically similar to *D.manhanensis*

**Table:** 

1	Leaf apices apically swollen as a propagulum	***D.anserinocapitatus***
–	Leaf apices not swollen, usually evenly narrowing	**2**
2	Cells on the upper ventral surface of the costa elongate	***D.wisselii***
–	Cells on the upper ventral surface of the costa quadrate	**3**
3	Laminal cells smooth	**4**
–	Laminal cells papillose	**10**
4	Costa with 2–3 layers of guide cells and without ventral stereids	**5**
–	Costa with 1 layer of guide cells and with ventral stereids	**6**
5	Leaves patent to spreading when moist, leaf lamina bistratose	***D.obtusus***
–	Leaves spreading when moist, leaf lamina unistratose	***D.manhanensis***
6	Costa percurrent or ending before the apex	**7**
–	Costa long-excurrent	**8**
7	Leaf margins bistratose near apex	***D.epapillatus***
–	Leaf margins unistratose	***D.mongolicus***
8	Plants flagellate, leaves linear-lanceolate	***D.ditrichoides***
–	Plants thickly leaved, leaves short-lanceolate to long-lanceolate	**9**
9	Leaves appressed when dry	***D.acutus***
–	Leaves twisted or incurved when dry	***D.validus***
10	Leaf margins plane	***D.tibeticus***
–	Leaf margins recurved	**11**
11	Costa without ventral stereids	**12**
–	Costa with ventral stereids	**13**
12	Costa excurrent	***D.daqingii***
–	Costa ending below apex	***D.imbricatus***
13	Marginal basal cells forming a distinctly differentiated area of smooth and transversely thick-walled cells	***D.hengduanensis***
–	Marginal basal cells not forming a distinctly differentiated area	**14**
14	Distal laminal cell superficial walls thicker than the internal walls	***D.mesopapillosus***
–	Distal laminal cell superficial walls of same thickness as the internal walls	**15**
15	Laminal cells with low papillae over the transverse walls, which reach the two adjacent cells	**16**
–	Laminal cells with papillae situated over the lumina	**17**
16	Leaves spreading when moist	***D.guangdongensis***
–	Leaves erect to patent when moist	***D.vulcanicus***
17	Leaf margins recurved in proximal 1/4–3/4	***D.icmadophilus***
–	Leaf margins strongly recurved or revolute to near apex	**18**
18	Leaf base squared in shape, costa slender	***D.tectorum***
–	Leaf base usually ovate in shape, costa stout	***D.cordatus***

## Supplementary Material

XML Treatment for Didymodon manhanensis
